# The mediating role of physical activity and health status between a health-supportive environment and well-being: a cross-sectional study

**DOI:** 10.3389/fpubh.2023.1233970

**Published:** 2023-10-19

**Authors:** Yi Liao, Xiaoyu Cheng, Zhuangzhuang Li, Yanyan Li

**Affiliations:** ^1^School of Mathematics and Statistics, Jiangxi Normal University, Nanchang, China; ^2^School of Physical Education, Wuhan Sports University, Wuhan, China; ^3^School of Sports Economics and Management, Hubei University of Economics, Wuhan, China

**Keywords:** environment and public health, built environment, social environment, well-being, physical activity, health status

## Abstract

**Objective:**

Based on the social-ecological systems theory and social support theory, this study aims to explore the relationship between a health-supportive environment and well-being among residents. It further examined the mediating role of physical activity and health status in the pathway between a health-supportive environment and well-being.

**Methods:**

The study utilized data from 2,717 samples of the China General Social Survey (2021) and conducted multiple regression analysis and mediation analysis using statistical software Stata 16.0 and SPSS PROCESS 3.3.

**Results:**

(1) A health-supportive environment had a significant impact on residents’ well-being (*t* = 8.476, *p* < 0.001). (2) Among the three dimensions of natural environment, built environment, and neighborhood social environment, the influence of neighborhood social relationship environment had the strongest influence on residents’ well-being (*t* = 8.443, *p* < 0.001). (3) Physical activity and health status played a mediating role in the relationship between a health-supportive environment and residents’ well-being. The mediating effect was as follows: health-supportive environment → physical activity → well-being with a mediation effect of 0.020; health-supportive environment → health status → well-being with a mediation effect of 0.029; health-supportive environment → physical activity → health status → well-being with a mediation effect of 0.008.

**Conclusion:**

A health-supportive environment not only directly influences residents’ well-being but also indirectly affects it through physical activity and health status. It is essential to focus on improving both the natural and built environment as well as the neighborhood social relationship environment in enhancing residents’ well-being. Physical activity serves as an important means to improve residents’ health level and promote their well-being.

## 1. Introduction

Well-being (WB) is a comprehensive emotional evaluation of an individual’s self-life state that reflects their perception of the meaning and goals of life, serving as an important indicator for assessing their quality of life (1). In China, “well-being” reflects people’s aspirations and pursuit of a better life, and this vision of happiness is closely linked to national development and construction (2). Since China’s reform and opening-up policy began in 1978, industrialization, urbanization and modernization have accelerated, the economy has rapidly grown, and the material living standards of Chinese citizens have continuously improved, resulting in a significant increase in happiness. However, with the continuous increase in economic investment and the consumption of natural resources, China’s economic development is facing serious environmental pollution. In addition, extensive urbanization has caused China’s urbanization scale to continue to expand, exacerbating the problem of imbalanced urban and rural development, leading to insufficient supply of public services in cities and relatively backward public services in rural areas, seriously hindering the improvement of residents’ WB in China (3). Especially in recent years, the phenomenon of stagnant or even declining WB levels among Chinese citizens has also attracted the attention of many scholars (4, 5), likely due to the rapid growth of China’s economy. And relevant research points out that the growth of WB in China may also face the “Easterlin paradox” (6). However, the level of economic development and income can only explain part of the effect on happiness levels. Multiple factors such as individual characteristics, social relationships, and the natural environment also have important impacts on individual WB (3).

In recent years, scholars from a range of disciplines, including sociology, geography, and urban planning, have paid increasing attention to the connection between the environment and WB. Particularly in the field of urban planning, the concept of a health-supportive environment has been increasingly emphasized. A health-supportive environment (HSE) refers to an environment that supports individuals’ health development during the process of health promotion, providing a healthy, safe, and happy living and working environment (7, 8). Such environments typically encompass multiple aspects, including natural, built, and social environments. As early as 1986, creating a HSE became one of the five strategies of the Ottawa Charter (9). In 2020, the World Health Organization identified the creation of health-supportive environments and the strengthening of the influence of health on WB as one of the four priority areas (10). Simultaneously, with the Chinese government’s increasing focus on public health, the “Outline of the fourteenth Five-Year Plan for National Economic and Social Development of the People’s Republic of China” was promulgated in 2022. The notice emphasizes the need to strengthen environmental construction, create health-supportive environments, promote residents’ health, and enhance their sense of WB (11). It can be concluded that creating a HSE has become an important approach for international organizations and government agencies to promote residents’ health and enhance their WB. In recent years, with the accelerated urbanization process in China, it is also facing social issues such as environmental concerns and urban development. In this context, the impact of a HSE on residents’ WB remains a topic worthy of research. How to create a favorable external environment and promote residents’ HSE is also a focal point in the field of urban development and related studies. Therefore, it is highly important to further investigate the relationship between a HSE and residents’ WB, as well as explore the influencing factors and pathways of residents’ WB. This will contribute to scientifically guiding the construction of the living environment in China and enhancing residents’ WB.

## 2. Literature review and theoretical hypotheses

### 2.1. The relationship between health-supportive environment and well-being

According to social-ecological systems theory, individual development is nested within a series of interrelated environmental systems, including microsystems, mesosystems, and macrosystems, which interact and influence each other, collectively affecting individual development (12). In the microsystem, individual behaviors and HS are important factors influencing residents’ WB (13). In the mesosystem, environmental systems such as the natural environment, built environment, and neighborhood social relationships are closely related to residents’ daily lives, constantly shaping individual development and potentially influencing residents’ WB (14). In addition, considering the HSE construction by organizations such as the World Health Organization (WHO) and the National Health Commission of the People’s Republic of China, the construction of HSE primarily involves multiple aspects, including the natural environment, built environment, and social environment, with the aim of promoting residents’ health and enhancing their WB through the optimization of these environmental factors. Combining social-ecological systems theory and policy documents, it can be inferred that the creation of HSE may indeed impact residents’ WB. Therefore, research has focused on elucidating the impact of the environment on WB from three dimensions: the natural environment, built environment, and social relationship environment (13, 15, 16).

Environmental pollution is a key factor that impacts residents’ WB at the natural environment level. Studies have shown that people are more concerned about issues such as air pollution, water pollution, and noise pollution in their daily lives, and the level of residents’ perception of pollution will affect their level of WB. The stronger the residents’ perception of environmental pollution is, the lower their level of WB will be (17, 18). In addition, the impact of environmental pollution on residents’ WB varies significantly among different population groups, with a more pronounced negative effect observed among individuals who are in poor health or belong to the older adult population (19). At the level of built environment, the relationship between built environment and residents’ WB has been a central focus in urban environmental planning and WB psychology due to the close connection between the residents’ daily lives and WB. Built environment mainly refers to various artificially constructed buildings or sites, such as public transportation systems, green spaces, pedestrian-friendly roads and well-equipped sports facilities (20–22). Cao, Ettema D., Schekkerman M., and other researchers have investigated the impact of urban built environments on residents’ WB, focusing on the 3D factors (density, design, and land-use mix) or 4D factors (design, diversity, public transit accessibility, and destination accessibility) of urban built environments (23–26). The research findings indicated that urban population density (24), the safety of the residential environment (27–29), well-designed communities (24), and accessibility to public transportation (30) can all influence residents’ WB. At the social relationship level, the neighborhood social relationship environment is an important way for individuals to connect with society, and studies have demonstrated that it has a significant impact on residents’ WB (31). Neighborhood social relationships, as a form of social capital for individuals, are powerful indicators for predicting WB (32). The closer the interaction between individuals and others is, and the more frequent the interaction in the neighborhood, the more WB can the individual experience (33). In conclusion, the natural environment, built environment, and social relationship environment can all have an impact on residents’ WB.

Based on the aforementioned research, the majority of studies have predominantly focused on the impact of single environmental factors on WB, particularly emphasizing the effects of environmental pollution and the built environment on residents’ WB. There has been relatively less attention given to the influence of neighborhood social relations on residents’ WB from a social relationship perspective. This overlooks the multidimensional impact of the environment on residents’ WB. According to the social-ecological systems theory (12), individuals’ lives and development are closely interconnected with the ecological systems in which they reside. At the micro-level, factors such as individual behaviors, socioeconomic environment, and educational attainment are important determinants of individual WB. At the meso-level, the overall environment of the community in which individuals reside (including natural environment, built environment, and neighborhood social relations) also plays a pivotal role in shaping individual WB. Therefore, it is plausible that the natural environment, built environment, and neighborhood social relations are all significant determinants of individual WB. Based on the above analysis, we propose Hypothesis 1:

*Hypothesis 1*: A health-supportive environment has a significant impact on residents’ well-being.

*Hypothesis 1a*: The natural environment has a significant impact on residents’ well-being.

*Hypothesis 1b*: The built environment has a significant impact on residents’ well-being.

*Hypothesis 1c*: The neighborhood social relationship environment has a significant impact on residents’ well-being.

### 2.2. The mediating role of physical activity

Engaging in physical activity (PA) has been found to help reduce the risk of cardiovascular diseases, including coronary heart disease and hypertension, and can effectively alleviate negative emotions such as depression and anxiety, which can contribute toward improvements in an individual’s mental health (34, 35). However, the level of individual engagement in PA is influenced by the external environment. According to social support theory, both instrumental support and emotional support are important sources of motivation for individuals to engage in PA (36, 37). Intrinsic emotional support refers to internal psychological factors, while instrumental support refers to external environmental factors. This argument seems to align with social-ecological theory, which suggests that individual behavior and activities are influenced by the external environment. Therefore, the study elucidates the influence of the environment on PA from three dimensions: the natural environment, the built environment, and the social relational environment.

At the level of the natural environment, environmental factors such as air pollution and noise pollution significantly impact individual engagement in PA. Moreover, higher levels of regional air pollution index are associated with lower frequencies of PA participation (38, 39). At the level of the built environment, the layout of public facilities, urban transportation systems, and road connectivity remain crucial areas of concern (40, 41). Studies have shown that the connectivity and accessibility of urban transportation, as well as the availability of public sports facilities, influence the frequency and duration of PA among residents. Convenient urban transportation and accessible public spaces can provide residents more opportunities to engage in PA and enhance their willingness to participate (40). In addition, WB is also an important factor that affects residents’ participation in physical activities. Neighborhood relationships are often an important indicator of community cohesion, and good neighborhood relationships mean that the individual’s community environment is more friendly, residents are more familiar with each other, and it is easier to find companions for physical activities, thereby promoting residents’ participation in physical activities (42). Neighborhood social relationships are a type of informal social capital, and studies have shown that high trust and high participation in social capital can significantly affect residents’ participation in physical activities (43, 44). However, in recent years, more and more researches have confirmed that PA can effectively improve individual WB. Actively participation in physical activities can effectively promote physical and mental health, boosting self-confidence, and thus improve an individual’s quality of life, and increase WB (14). Based on the above analysis, hypothesis 2 is proposed:

*Hypothesis 2*: Physical activity plays a mediating role in the health-supportive environment and well-being.

### 2.3. The mediating role of health status

Public health issues have always been a key topic of concern for governments, citizens, and researchers, and a HSE is the cornerstone of promoting public health (13). Studies have shown that long-term residence in environments with severe air pollution can lead to an increase in respiratory and cardiovascular diseases, severely affecting residents’ physical health. On the other hand, residents who live in environments with good living conditions are generally healthier both psychologically and physically (45, 46). In addition, the built environment of cities is also one of the most important potential factors contributing to health inequality among residents (47). Elements of the built environment such as building density, urban transportation, and public service facilities can all have an impact on residents’ health. However, health is also an important factor affecting residents’ WB (48, 49). According to Bussiere et al., health status (HS) significantly affects an individual’s WB, and as age increases, the effect of health on WB becomes stronger (48). Reducing adverse HS helps individuals achieve WB (48). HS refers to a state beyond simply being free from disease, where the body, mind, and social relationships are all in good condition. According to evidence from neuroscience and psychology (50, 51), when an individual’s mental and physical health are both in good condition, the central nervous system in the brain is also in a stable and balanced state, enabling better psychological responses to different stimuli in life and better acquisition and accumulation of WB (51, 52). Based on the above analysis, it can be observed that changes in the external environment can influence residents’ HS, which in turn impact their attainment of subjective WB. Therefore, Hypothesis 3 is proposed:

*Hypothesis 3*: Health status plays a mediating role in the relationship between a health-supportive environment and well-being.

### 2.4. Chain mediating role of physical activity and health status

PA is an effective way to improve individual health, relieve mental stress, and promote WB. This conclusion has been widely accepted and confirmed (53). The main mechanism is that appropriate PA can enhance the brain’s sensitivity to serotonin and norepinephrine, thereby individual depression and anxiety could be released, and psychological health be promoted (53). Physiologically, long-term moderate PA can effectively promote blood circulation, reduce blood pressure and lipid levels, and reduce the risk of cardiovascular and cerebrovascular diseases (54, 55). However, with the acceleration of urbanization and industrialization, the impact of supportive environmental factors such as natural environment, built environment, and neighborhood social relationship environment on residents’ participation in PA has become increasingly important. Factors such as air pollution, transportation systems, and green areas can all affect residents’ participation in PA (56). The reduction of PA can lead to individual obesity, increased risk of cardiovascular and cerebrovascular diseases, lower levels of individual health, and hinder the attainment and improvement of individual WB (57). Indeed, it can be inferred that PA is highly likely to impact residents’ subjective WB through their HS. Based on the above analysis, Hypothesis 4 is proposed:

*Hypothesis 4*: Physical activity and health status play a chain-mediated role between a health-supportive environment and well-being.

**Figure 1:**
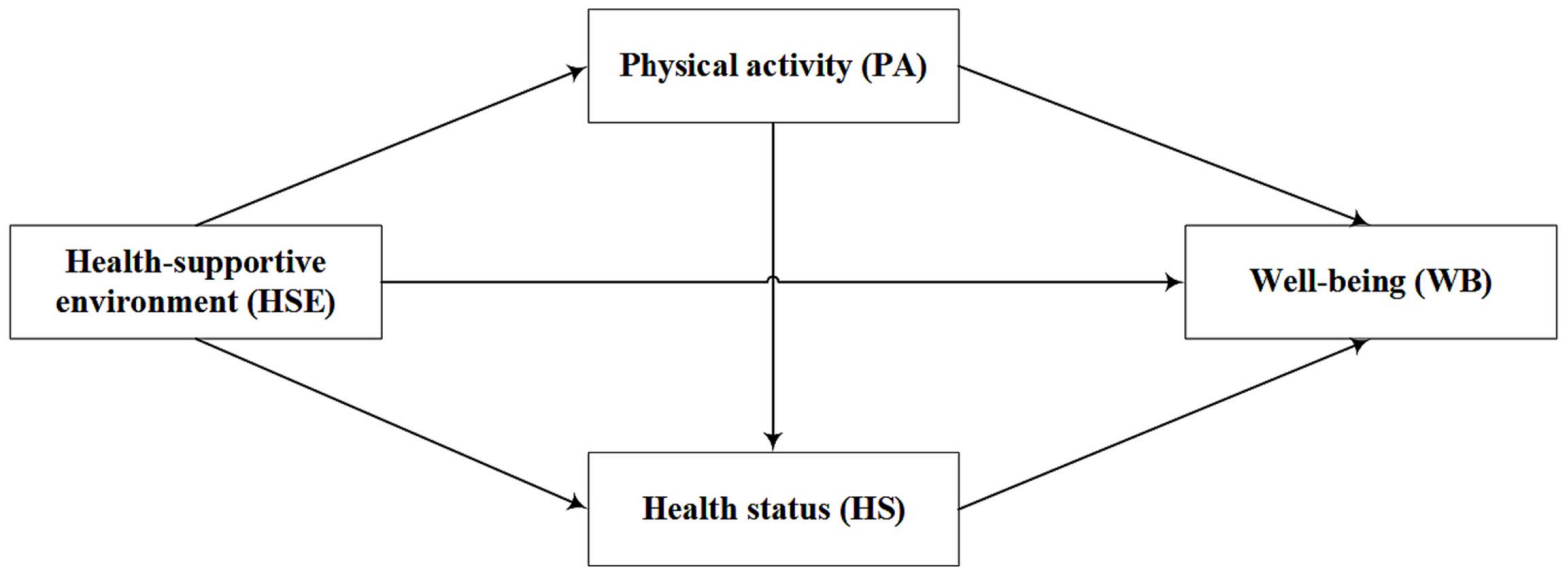
The hypothetical structure model.

Based on the above analysis, this study used data from the China Social Survey in 2021 to explore the impact of a HSE on residents’ WB (8), and further explored the mediating role of PA and HS in the relationship between a HSE and WB. The study aimed to provide theoretical support and practical guidance for promoting residents’ WB (Figure 1).

## 3. Materials and methods

### 3.1. Material source

The data used in this study was obtained from the 2021 Chinese General Social Survey (CGSS 2021), which is the first comprehensive nationwide social survey project in China. The survey was conducted collaboratively by various academic institutions across the country, with Renmin University of China as the lead institution. A multi-stage stratified random sampling method was employed to extract samples from 28 provinces, municipalities, and autonomous regions in China, and collect data systematically on individuals, households, and communities at multiple levels. The CGSS (2021) survey questionnaire mainly consists of three parts: the core module, thematic modules, and add-on modules. The core module includes social demographic characteristics, health, class identity, and other content; the thematic modules include topics such as COVID-19 pandemic and work and occupation; and the add-on modules include the East Asian Social Survey (EASS) health module, the International Social Survey Program (ISSP) health module, and the ISSP environmental module. The sample size for the core and thematic modules is 8,000, which includes all survey respondents, while the sample size for the additional modules is 2,717, which are randomly drawn from one-third of the total sample. As the selected variables in this paper involves the add-on modules, and there were no missing values for HSE, happiness, PA, and other survey questions, but there were missing values for some control variables (such as economic status and marital status). Therefore, the median imputation method was used to impute some control variables, resulting in a total of 2,717 valid samples.

### 3.2. Variables

The independent variable studied was the health-supportive environment (HSE). Referring to previous research and combining with the CGSS survey questionnaire, the study mainly measured the HSE from three parts: natural environment, built environment, and neighborhood social relationship environment. On the natural environment the three items of the “CGSS” theme module survey were used: How serious are the problems of *“air pollution,” “water pollution,” and “noise pollution” in the place where you live?* The items were assigned values from 1 to 4, with 1 indicating very serious and 4 indicating not at all serious. The higher the value was, the less residents thought there was air pollution, water pollution, and noise pollution in their local area. The Cronbach’s coefficient of this dimension was 0.750. The built environment was measured with the following four items: To what extent do you agree with the following statements: *“Within one kilometer (about a 15-min walk) around your home, there are suitable places for sports such as jogging and walking,” “Within one kilometer (about a 15-min walk) around your home, there are many fresh vegetables and fruits to choose from,” “Within one kilometer (about a 15-min walk) around your home, there are sufficient public facilities such as community centers, libraries, parks, etc.,”* “*The place where I live is very safe*.” The items were measured with a five-point Likert scale ranging from 1 (completely agree) to 5 (completely disagree). Finally, the study reversed the scores of these four items to obtain the score for measuring the built environment. The Cronbach’s coefficient of this dimension was 0.732. The neighborhood (social relationship) environment was mainly measured with the following two items: To what extent do you agree with the following statements: “*Neighbors around me care for each other*” and “*When I need help, my neighbors are willing to help me*.” The items were measured with a five-point Likert scale ranging from 1 (completely agree) to 5 (completely disagree). Finally, the study reversed the scores of these two items to obtain the score for measuring the neighborhood social relationship environment. The Cronbach’s coefficient of this dimension was 0.860. The study added up and averaged the scores of the items in these three parts to obtain the score for the HSE.

The dependent variable of the study was well-being (WB). Referring to previous research (58), the study measured residents’ self-assessed WB, using the question “*Do you feel happy with your life?*” and responses were measured on a five-point Likert scale ranging from 1 (not at all happy) to 5 (extremely happy).

The mediating variable in the study is physical activity (PA). Based on existing research (59), physical activity was assessed using the item “Frequency of engaging in sports exercises during leisure time in the past year” from the “CGSS” survey. The item was rated on a scale of 1 to 5, where 1 = daily, 2 = several times a week, 3 = several times a month, 4 = a few times a year or less, and 5 = never. Finally, reverse scoring was applied to the item.

Another mediating variable is health status (HS). The research utilized self-rated health assessment by the residents to measure their health status. Self-rated health was measured using the following three items: “How would you rate your current physical health?,” “In the past four weeks, how much did physical health problems interfere with your usual activities, including work or daily tasks?,” “In the past four weeks, to what extent have you felt depressed or downhearted?.” These items were measured using a Likert scale with four levels, where higher values indicate better health status. The Cronbach’s alpha coefficient for this scale was 0.747. To obtain a health status score for each resident, the scores of the three items were summed and averaged.

### 3.3. Control variables

According to existing studies, we also included control variables such as gender, urban–rural status, age, education level, family economic status, social class identity, marital status, COVID-19 risk perception, and provincial location (Table 1).

**Table 1:** Definition and assignment of control variables.

Variables	Variable description
Gender	Male = 1, Female = 0
Urban and rural	Urban = 1, Rural = 0
Age	According to the Chinese national age classification standards, the age of the population is divided into three stages: youth (18–39 years old) = 1, middle age (40–65 years old) = 2 and older adult (66 years old and above) = 3. Using the older adult as a reference, virtualization
Education level	No education = 0, elementary education = 6, middle school education = 9, high school education = 12, college = 15, bachelor’s degree = 16, graduate and above = 19
Family economic status	Below average = 1, average = 2, above average = 3. Virtualization with below-average as reference
Social class identity	The minimum value is 1, the maximum value is 10, the larger the value means the higher the class
Marital status	Spouse and cohabitation = 1, other (unmarried, widowed) = 0
COVID-19 risk perception	How likely do you think you are to be infected with New Crown? Extremely unlikely = 1, Extremely likely = 7
Provincial location	East = 1, Central = 2, West = 3. Virtualization with the western region as a reference

### 3.4. Analytic approach

The study is a cross-sectional research that utilized Stata 16.0 for descriptive analysis, correlation analysis, and multiple regression analysis. Descriptive statistics, including percentages, means, and standard deviations, were employed to analyze all variables in the study. Correlation analysis was conducted to measure the relationships between HSE, PA, HS, and WB. The significance level for statistical analysis was set at *p* < 0.005 (two-tailed test). Multiple regression analysis was used to explore the relative contributions of HSE and control variables to WB. Finally, the study employed SPSS PROCESS 3.3 to examine the mediating effect of PA and HS in the relationship between HSE and WB (60).

In order to meet the assumption of normality for the residuals in multiple linear regression analysis, it is necessary to test whether the dependent variable follows a normal distribution. The study used the Shapiro-Wilks method to test the normality of the three variables: PA, HS and WB levels. The data results indicated *p* < 0.001, suggesting that all three variables exhibited a normal distribution, thereby satisfying the assumption of normality for the residuals in multiple linear regression analysis.

## 4. Results and analysis

### 4.1. Common method bias

To avoid the potential common method bias caused by self-evaluation methods and negatively scored items, this study employed the Herman single-factor test method to test for common method bias. The results of the test showed that three factors were extracted from the unrotated exploratory factor analysis with eigenvalues greater than 1, and the maximum variance explained by a single factor was 35.487% (less than 40%). Therefore, this study does not have a serious problem of common method bias.

### 4.2. Descriptive statistics

Table 2 presents the descriptive statistics of the variables studied. Females account for 54.8% of the total sample. Regarding the age distribution, the youth, middle-aged, and older adult represent 24.7, 33.5, and 41.8% of the total sample, respectively. In terms of the urban–rural distribution, urban residents account for 55.9% of the total sample. The average educational level of the survey respondents is at the secondary school level. Additionally, it is worth noting that the mean value of neighborhood relationships among residents is very high, much higher than the overall average. Regarding residents’ WB, 57.2% of survey respondents believe that they are happy.

**Table 2:** Descriptive statistics.

Variable	Mean/Ratio	S.D.	Min	Max
Natural environment	3.041	0.653	1	4
Built environment	3.700	0.762	1	5
Neighborhood social relations environment	4.047	0.643	1	5
Health-supportive environment	12.802	3.075	3	17
Well-being	3.980	0.882	1	5
Very unhappy	1.4%			
More unhappy	4.5%			
I cannot say if I’m happy or not	12.8%			
Happier	57.2%			
Very happy	24.2%			
Physical activity	3.830	1.620	1	5
Health status	3.776	0.918	1	5
Gender	0.452	0.498	0	1
Female	54.8%			
Male	45.2%			
Age	–	–	1	3
Youth	24.7%			
Middle age	33.5%			
Older adult	41.8%			
Urban and rural	0.559	0.496	0	1
City	55.9%			
Rural	44.1%			
Education level	9.251	4.742	0	19
Family economic status	–	–	1	3
Below average	39.5%			
Average	52.7%			
Higher than flat level	7.9%			
Social class	4.29	1.890	1	10
Marriage	0.741	0.438	0	1
Spouse	74.1%			
Other	25.9%			
COVID-19 risk perception	5.60	1.612	1	7
Provincial location	–	–	1	3
East	40.4%			
Middle	33.6%			
West	26.0%			

S.D., standard deviation; Min, minimum; Max, maximum.

### 4.3. Correlation analysis

Table 2 displayed the results of the correlation analysis for the independent variables, dependent variables, and mediator variables. The Spearman correlation results indicated significant positive correlations between subjective WB and HSE (*r* = 0.187, *p* < 0.001), PA and HSE (*r* = 0.072, *p* < 0.001), as well as WB (*r* = 0.158, *p* < 0.001). Additionally, there were significant positive correlations observed between HS and HSE (*r* = 0.088, *p* < 0.001), WB (*r* = 0.0268, *p* < 0.001), and PA (*r* = 0.170, *p* < 0.001, Table 3).

**Table 3:** Correlation analysis.

Variable	A	B	C	D
A. Health-supportive environment (HSE)	–			
B. Well-being (WB)	0.187^**^	–		
C. Physical activity (PA)	0.072^**^	0.158^**^	–	
D. Health status (HS)	0.088^**^	0.268^**^	0.170^**^	–

^**^Correlations are significant at the 0.001 level (two-tailed).

### 4.4. Regression analysis

Model 1–3 reported the impact of natural environment, built environment, and neighborhood social relations environment on residents’ WB. The regression results showed that all three environments significantly affected residents’ subjective WB, that is, the better the conditions of natural environment, built environment, and neighborhood social relations environment are, the stronger residents’ WB will be (see Table 4). Hypotheses 1a, 1b, and 1c were confirmed. Among them, the neighborhood social relations environment had the greatest impact on residents’ WB. Model 4 reported the impact of HSE on residents’ WB. The results showed that the HSE significantly affected residents’ WB. That is, as the overall improvement of the HSE, residents’ WB will also increase accordingly. Hypothesis 1 was confirmed.

**Table 4:** Regression analysis.

Variable	Model 1	Model 2	Model 3	Model 4
Natural environment	3.495^***^			
	(0.067)			
Built environment		5.546^***^		
		(0.106)		
Neighborhood social relations environment			8.443^***^	
			(0.161)	
Health-supportive environment (HSE)				8.476^***^
				(0.159)
Gender	0.824	0.909	1.085	0.889
	(0.016)	(0.017)	(0.020)	(0.017)
Age stage (with reference to old age)				
Youth	−3.155^***^	−3.287^***^	−2.653^**^	−2.661^**^
	(−0.086)	(−0.087)	(−0.070)	(−0.070)
Middle youth	−4.948^***^	−5.135^***^	−4.825^***^	−4.966^***^
	(−0.116)	(−0.120)	(−0.112)	(−0.115)
Urban and rural	0.575	−0.846	1.294	0.683
	(0.012)	(−0.018)	(0.027)	(0.014)
Education level	2.601^**^	2.120^*^	2.893^**^	2.661^**^
	(0.063)	(0.052)	(0.070)	(0.064)
Family economic status (using below average as a reference)				
Average	6.519^***^	6.424^***^	6.678^***^	6.323^***^
	(0.137)	(0.134)	(0.138)	(0.130)
Above average	4.280^***^	4.183^***^	4.590^***^	4.233^***^
	(0.088)	(0.086)	(0.093)	(0.086)
Social class identity	10.892^***^	10.488^***^	10.209^***^	10.154^***^
	(0.216)	(0.208)	(0.202)	(0.201)
Marital status	3.588^***^	3.520^***^	3.436^***^	3.489^***^
	(0.070)	(0.069)	(0.067)	(0.068)
COVID-19 risk perception	2.958^**^	3.153^***^	2.559^*^	2.514^*^
	(0.055)	(0.058)	(0.047)	(0.046)
Provincial location (with reference to the west)				
East	2.958^**^	2.870^**^	3.436^**^	3.229^**^
	(0.055)	(0.069)	(0.067)	(0.077)
Middle	0.724	0.621	0.639	0.642
	(0.017)	(0.014)	(0.015)	(0.015)
F	28.623	30.250	33.806	39.967
R^2^	0.127	0.134	0.147	0.147
△R^2^	0.123	0.129	0.143	0.143
Constant	38.928^***^	39.485^***^	39.608^***^	39.967^***^

^*^ indicates *p* < 0.05, ^**^ indicates *p* < 0.01, ^***^ indicates *p* < 0.001; values in parentheses are standard errors.

In the analysis of factors influencing residents’ WB, various factors such as age, education level, family economic status, social class identity, and marital status all had an impact on residents’ WB. Specifically, in terms of age, compared with the elder the WB of the young and middle-aged was lower. Regarding education level, there was a positive relation with level of WB. In terms of family economic status, residents with average or above-average family economic status tended to have significantly higher WB than those with below-average economic status. Additionally, higher social class identity was associated with stronger WB among residents. Unmarried and widowed individuals tended to have lower WB compared to married individuals. At the regional level, there were significant regional differences in residents’ WB in China, with residents in eastern regions exhibiting significantly higher WB than those in western regions.

Furthermore, the data for this study was obtained in 2020, a period when the COVID-19 virus was rampant in China, thus the study was conducted with a special social context. Therefore, the study also examined the impact of COVID-19 risk perception on residents’ WB. The data results indicated that residents with stronger perception of COVID-19 risks tended to have stronger WB. This data result may be related to the epidemic prevention measures adopted by China. In 2020, with the global outbreak of the pandemic, the Chinese government implemented strict measures to control the spread of the virus, including restricting large-scale population movements and reducing social interactions. These measures was helpful to control the spread of the epidemic increased residents’ risk perception to a certain extent and prevented widespread infection. After implementing strict control measures, China effectively controlled the situation, maintaining a stable number of infected individuals, which potentially contributed to an improvement in residents’ WB to some extent.

### 4.5. Mediation chain analysis

Table 5 reports the path coefficients of the mediation model, where COVID-19 Risk Perception is included as a control variable in the mediation model. Table 6 reported the direct effect value of HSE on WB, the total mediation effect value of the mediation model, and the mediation effect values of the three indirect paths. The results showed that the direct effect of HSE on residents’ WB was 0.106 [95% CI: 0.069, 0.143], and Hypothesis 1 was confirmed. The results of the three indirect paths analysis showed that Path 1 health-supportive environment→physical activity→well-being had a mediation effect of 0.020 [95% CI: 0.012, 0.029], and Hypothesis 2 was confirmed. Path 2 health-supportive environment→health status→well-being had a mediation effect of 0.029 [95% CI: 0.016, 0.038], and Hypothesis 3 was confirmed. Path 3 health-supportive environment→physical activity→health status→well-being had a mediation effect of 0.008 [95% CI: 0.005, 0.010], and Hypothesis 4 was confirmed.

**Table 5:** Regression analysis of the chained mediation model between physical activity and health status.

Variable		Model index			*β*	CI		*t*
Outcome variable	Predictor variable	*R*	*R* ^2^	*F*		Boot LLCI	Boot ULCI	
Physical activity	Health-supportive environment	0.214	0.046	64.810^***^	0.211	0.174	0.247	11.208^***^
	COVID-19 risk perception				0.030	−0.005	0.065	1.710
Health status	Health-supportive environment	0.213	0.045	42.703^***^	0.113	0.677	0.137	5.795^***^
	Physical activity				0.146	0.099	0.168	7.565^***^
	COVID-19 risk perception				0.053	0.021	0.086	3.266^*^
Well-being	Health-supportive environment	0.311	0.097	72.364^***^	0.106	0.069	0.143	5.647^***^
	Physical activity				0.097	0.060	0.134	5.126^***^
	Health status				0.258	0.217	0.297	12.639^***^
	COVID-19 risk perception				0.011	−0.023	0.045	0.066

^*^ indicates *p* < 0.05, ^**^ indicates *p* < 0.01, ^***^ indicates *p* < 0.001.

**Table 6:** Mediation effect analysis.

	Effect	BootSE	Boot LLCI	Boot ULCI
Path 1	0.020	0.004	0.012	0.029
Path 2	0.029	0.006	0.016	0.038
Path 3	0.008	0.001	0.005	0.010
Direct effect	0.106	0.019	0.069	0.143
Total effect	0.163	0.019	0.124	0.198

Path 1: health-supportive environment→ physical activity→ well-being; Path 2: health-supportive environment→ health status→ well-being; Path 3: health-supportive environment→physical activity→ health status→ well-being.

## 5. Discussion

Based on Social Ecology Theory and Social Support Theory, this study explored the impact of a HSE on residents’ WB, with PA and HS as mediators. The study examined the impact of three dimensions of a HSE, namely natural environment, built environment, and neighborhood social relationship environment, on residents’ WB.

The results of the study showed that a HSE significantly predicts residents’ WB, and the natural environment, built environment, and neighborhood social relationship environment all has an impact on residents’ WB, which is consistent with previous research (17, 18, 31). Regarding the impact on residents’ WB, there exists significant disparities among the natural environment, built environment, and social-neighborhood relationship environment. The neighborhood social relationship environment has the greatest impact on residents’ WB, explaining 8% of the impact effect, while the natural environment has the smallest impact, explaining only 3% of the impact effect. This indicated that the microsystem of the neighborhood social relationship environment is more important in influencing residents’ WB than the mesosystem of the natural environment and the built environment. This study also confirmed the “Seven” influencing factors of WB and the findings of related fields in psychology, indicated that micro factors such as the neighborhood social relationship environment are important to residents’ WB (26), while factors such as the natural environment have a relatively small impact (52). In addition, in terms of the total effect, a HSE also has a significant impact on residents’ WB, explaining 8% of the impact effect. Compared with the built environment, a HSE has a greater total effect, which is a phenomenon worthy of consideration. The study speculated that the impact of the built environment on residents’ WB may be influenced by work type and working hours. When individuals work longer hours or engage in heavy physical labor, they are more likely to be in a state of physical and mental fatigue, and they may have less time for leisure and outdoor activities, thus reducing the impact of the built environment on residents (61, 62).

The results of mediation analysis in path 1 showed that PA plays a partial mediating role between a HSE and residents’ WB, which is consistent with previous research (63, 64). Based on the Social Ecology Theory and Social Support Theory, the external environment and social support are important factors that influence individual behavior and emotions. The natural environment, built environment, and neighborhood social relationship environment can all affect the frequency and time of residents’ participation in physical activities. A good natural and built environment can increase residents’ willingness to participate in outdoor physical activities (40, 65), while good social relationships can help residents find companions to participate in physical activities in their community (43). Under the dual effects of environment and emotion, a good HSE can help improve the frequency and time of residents’ physical activities, thereby effectively enhancing residents’ WB.

The results of mediation analysis in path 2 showed that HS plays a partial mediating role between a HSE and residents’ WB, which is consistent with previous research (52). Based on the Social Ecology Theory, micro, meso and macro environments where individuals lived can have an impact on themselves. In terms of health effects, environmental pollution in the natural environment may increase disease risk and harm residents’ physical and mental health (66, 67); factors such as the completeness of public facilities and land use intensity in the built environment are directly related to residents’ quality of life, and a decline in their quality of life will inevitably affect residents’ physical and mental health (68). Good social relationships can help residents better integrate into society, obtain social support, and promote individual health (69). Under the support of the meso natural environment, micro built environment, and neighborhood social relationship environment, individual qHS can be improved, thereby promoting residents’ WB.

The analysis results of chain mediation in path 3 suggested that PA and HS mediate the relationship between a HSE and residents’ WB. From a social psychology perspective, an individual’s WB could be influenced by multiple factors at various levels, including external environmental factors and individual characteristics. PA and HS are important pathways through which a HSE affects residents’ WB. Firstly, PA is an important way to promote residents’ physical and mental health. Positive and reasonable PA can effectively alleviate stress in daily life, relieve negative emotions such as depression and anxiety, enhance physical fitness, reduce the risk of individual cardiovascular diseases, and thereby improve individual HS (53, 54). The improvement in HS helps individuals experience life, feel happy, and thus enhance their WB (51). In addition, the natural environment, built environment, and neighborhood social relationship environment in a HSE can all affect residents’ participation frequency or time in PA. Long-term adherence to appropriate PA can effectively improve individual HS (70). Residents who maintain good HS in the long term can maintain an optimistic and positive attitude in life and work, take the initiative to participate in social activities, enjoy life, and experience WB (71). At this point, under the increasingly perfect background of the development of a HSE, individuals’ WB will develop in a good direction. Therefore, a HSE affects residents’ WB through PA and HS.

In the analysis of mediating effects, it was worth noting that the mediating effect of the chain path involving PA and HS was relatively small, while the direct effect of a HSE on WB was larger. In terms of path coefficients, the main reason for this was that the impact of PA on residents’ health had a relatively small effect, which differed significantly from existing research. Considering the actual social context at the time of data collection, it was speculated that there were two main reasons for this phenomenon. Firstly, the data collection took place in 2021, during the global COVID-19 pandemic, and residents’ participation in PA and HS may have been influenced by the novel coronavirus (72). Secondly, it was influenced by China’s epidemic prevention and control policies. During the outbreak of COVID-19, the Chinese government implemented measures to protect residents from infection, aiming to prevent the wide spread of the virus (73). These measures may indirectly affect residents’ participation in PA. The decline in PA among residents, coupled with the impact of the COVID-19 pandemic on their physical and mental health, as well as the severe economic downturn, had increased individual pressures in daily life and work. This had led to an increase in physical and mental health issues among individuals, resulting in a decreased impact of PA on residents’ HS (74, 75).

## 6. Conclusion

The study explored the impact of a HSE on residents’ WB, and how a HSE affects residents’ WB through PA and HS. The study found that a HSE has a positive effect on residents’ WB, and can affect residents’ WB through PA and HS. Furthermore, the study compared the effects of natural environment, built environment, and neighborhood social relationship environment on residents’ WB, and confirmed that the neighborhood social relationship environment, as a micro-level system, had a greater impact on residents’ WB. These findings contributed to a better understanding of the relationship between a HSE and residents’ WB, providing reference suggestions for guiding the construction of residential environments in China. Based on the results, in the process of creating a HSE we should not only focus on improving the natural and built environments but also emphasize the influence of the neighborhood social relationship environment. Additionally, measures should be taken to promote residents’ participation in PA, enhance their health levels, and thereby improve residents’ WB. Moreover, the study incorporated the perception of COVID-19 risk as an important influencing factor in the model, which partly explained its impact on residents’ WB. Finally, the study explored the mediating effects of PA and HS on the relationship between a HSE and residents’ WB, providing important theoretical support for promoting residents’ participation in PA, improving their health levels, and enhancing their WB.

## 7. Limitation

The study also has some limitations. First, because the data source is a public database, the built environment was measured only in terms of the proximity and safety of community public facilities, which is not comprehensive compared to the 3D or 4D factors. Second, in the article, we did not consider the bidirectional causal relationship between health and WB, as some studies have shown that individual WB may also have an impact on health (49, 76). However, due to the cross-sectional nature of the data, it is not possible to use time-series methods to eliminate the influence of bidirectional causality. In future research, it would be beneficial to employ specialized scales to measure the multi-dimensional environmental systems and compare their effects on residents’ WB. In terms of research design, longitudinal survey data can be used to further elucidate the impact of different types of environments on residents’ WB. Lastly, considering the unique context in which the data was collected in this survey, future studies can explore the effects of PA on HS, taking into account the specific social background during data collection.

## Data availability statement

The original contributions presented in the study are included in the article/supplementary material, further inquiries can be directed to the corresponding author.

## Author contributions

YL: conceptualization, manuscript writing, and data analysis. XC: revision and proofreading. ZL: translation and revision. YYL: manuscript writing and revision. All authors participated in the article and approved the submitted version.
